# Low frequency deep brain stimulation of nucleus accumbens shell neuronal subpopulations attenuates cocaine seeking selectively in male rats

**DOI:** 10.1016/j.addicn.2023.100133

**Published:** 2023-10-20

**Authors:** Sarah E. Swinford-Jackson, Matthew T. Rich, Phillip J. Huffman, Melissa C. Knouse, Arthur S. Thomas, Sharvari Mankame, Samantha J. Worobey, R. Christopher Pierce

**Affiliations:** a Brain Health Institute and Department of Psychiatry, Rutgers Robert Wood Johnson Medical School, Piscataway, NJ 08854 USA; b Center for Neurobiology and Behavior, Perelman School of Medicine, University of Pennsylvania, Philadelphia, PA 19104 USA

**Keywords:** Deep brain stimulation, Nucleus accumbens, Cocaine relapse, Optogenetics, LTP

## Abstract

The present study examined the effect of deep brain stimulation (DBS) in the nucleus accumbens shell on cocaine seeking and neuronal plasticity in rats. Electrical DBS of the accumbens shell attenuated cocaine primed reinstatement across a range of frequencies as low as 12 Hz in male rats. Nucleus accumbens medium spiny neurons (MSNs) can be differentiated by expression of dopamine D1 receptors (D1DRs) or D2DRs. Low-frequency optogenetic-DBS in D1DR- or D2DR-containing neurons attenuated cocaine seeking in male but not female rats. In slice electrophysiology experiments, 12 Hz electrical stimulation evoked long term potentiation (LTP) in D1DR-MSNs and D2DR-MSNs from cocaine naive male and female rats. However, in cocaine-experienced rats, electrical and optical DBS only elicited LTP in D2DR-MSNs from male rats. These results suggest that low frequency DBS in the nucleus accumbens shell effectively, but sex-specifically, suppresses cocaine seeking, which may be associated with the reversal of synaptic plasticity deficits in D2DR-MSNs.

## 1. Introduction

High rates of relapse remain a significant impediment to recovery in individuals with cocaine use disorder [1]. Although effective pharmacological therapies are unavailable, DBS has been established as a treatment possibility for severe substance use disorders [2]. First approved for Parkinson’s Disease, clinical studies using DBS now include other neurological and neuropsychiatric disorders including substance use disorders [2-5]. Early clinical and preclinical studies on Parkinson’s Disease informed commonly used stimulation parameters, typically characterized by higher stimulation frequencies (i.e., greater than 100 Hz) [6,7]. Recent studies have begun to explore the therapeutic benefit of lower frequency DBS [8-11], although no clinical studies have yet applied these parameters to substance use disorder treatment.

Facets of cocaine use disorder can be modeled in rodents using drug self-administration paradigms. In rats, bilateral high frequency DBS of the nucleus accumbens shell attenuated cocaine priming- and cue-induced reinstatement of cocaine seeking [12-14], two common models of relapse. In contrast, in rats which expressed escalated cocaine self-administration, accumbens DBS actually increased cocaine taking [15]. Preclinical DBS studies [12-14,16] have typically employed the high frequency parameters most often used clinically [2,17-22]. However, low frequency accumbens DBS has also been shown to alter drug-related behaviors. For example, chronic unilateral nucleus accumbens shell DBS delivered at 20 Hz during abstinence from cocaine self-administration attenuated cocaine seeking [23].

The vast majority of accumbens neurons are medium spiny efferent neurons. These medium spiny neurons (MSNs) are all GABAergic but can be divided into two similar sized populations based on the expression of either D1DRs or D2DRs, although there is co-expression in a limited number of MSNs [24]. Chemogenetic and optogenetic approaches have generally defined bidirectional roles for accumbens D1DR-MSNs and D2DR-MSNs such that activation of D1DR-MSNs potentiates various cocaine-mediated behaviors, whereas they are inhibited by stimulation of D2DR-MSNs [25-27]. However, recent work suggests D1DR-MSNs and D2DR-MSNs can each control reward and aversion under different stimulation parameters, indicating that this dichotomy may be condition-dependent [28]. Recent studies have begun to explore the cell subtype-specificity of DBS to modulate behavior using optogenetic approaches. Low frequency opto-DBS (12 Hz) of neurons projecting from the prefrontal cortex onto accumbens D1DR-MSNs blocked cocaine-evoked locomotor sensitization and altered long-term synaptic plasticity; importantly, these effects were only elicited by 12 Hz electrical DBS when administered in combination with a D1DR antagonist [29]. Mimicking high frequency electrical DBS with optogenetic stimulation within nucleus accumbens shell neuronal subtypes indicated that 130 Hz opto-DBS of D2DR-containing, but not D1DR-containing neurons attenuated cocaine seeking in male rats [30]. Interestingly, high frequency opto-DBS of D1DR- and D2DR-containing neurons did not alter cocaine seeking in female rats, highlighting a potential sex difference in the efficacy of stimulation approaches [30]. How DBS and opto-DBS alter synaptic physiology to influence cocaine seeking in cocaine-experienced rats is not yet clear. These studies suggest that MSN subtype-specific manipulation using opto-DBS may be used to disentangle the intricate and perhaps even opposing effects of electrical DBS which impinge on cocaine-related behaviors.

The present study expanded on our prior work with high frequency stimulation and aimed to investigate the effect of low frequency accumbens shell DBS on cocaine seeking and synaptic plasticity. We focused on the nucleus accumbens shell given that high frequency electrical DBS attenuated cocaine seeking when administered to the shell but not the core subregion of the nucleus accumbens [13]. Here, we show that nucleus accumbens shell DBS attenuated cocaine seeking over a range of frequencies in male rats, including frequencies as low as 12 Hz. We then tested the hypothesis that, similar to high frequency stimulation, low frequency opto-DBS of D2DR-containing, but not D1DR-containing neurons, in the accumbens shell suppresses cocaine seeking. DBS was mimicked within specific MSN subtypes by combining low frequency optogenetic stimulation in D1DR-Cre and D2DR-Cre rats [30-33]. Experiments were performed in male and female rats to explore whether low frequency opto-DBS elicits a sex-specific effect on cocaine seeking, similar to what we previously observed with high frequency stimulation [30]. To parse out the effect of electrical and optical DBS on long term synaptic plasticity within accumbens MSN subtypes, slice electrophysiology recordings were performed in naïve and cocaine-trained, male and female, D1DR-Cre and D2DR-Cre rats.

## 2. Materials and methods

### 2.1. Animals and housing

Male Sprague Dawley rats (250–300 g) were procured from Taconic (Germantown, NY). LE-Tg(Drd1a-iCre)3Ottc (RRRC#:00767; D1DR-Cre) and LE-Tg(Drd2-iCre)1Ottc (RRRC#:00768; D2DR-Cre) male founders were generated by the NIDA Optogenetics and Transgenic Technology Core (now Transgenic Rat Project, NIDA IRP, Bethesda, MA) and obtained from the Rat Resource and Research Center (RRRC, Colombia, MS). Female Long Evans breeders were obtained from Charles River Laboratories (Wilmington, MA) and lines were backcrossed frequently to prevent genetic drift. Adult (PND 60–240) male and female Long Evans transgenic D1DR-Cre or D2DR-Cre rats used in experiments were bred in house and Cre expression in D1DR- or D2DR-containing neurons was confirmed by genotyping with the following primers (5′ to 3′): D1DR forward - CTC CTG ATG GAA CCC TAC CA; D2DR forward - TCA GGG AAC CCT CTT TGA GA; Cre reverse - CAC AGT CAG CAG GTT GGA GA (Sigma Aldrich, St. Louis, MO). Rats were individually housed with food and water available *ad libitum*. A 12/12 hr light/dark cycle was used with the lights on at 6:00 a.m. All experimental procedures were performed during the light cycle. All experimental procedures were consistent with the ethical guidelines of the US National Institutes of Health and were approved by the Institutional Animal Care and Use Committee.

### 2.2. Drugs

Cocaine hydrochloride was obtained from the National Institute on Drug Abuse (Rockville, MD) and dissolved in bacteriostatic 0.9 % saline.

### 2.3. Surgery

Prior to surgery, rats were anesthetized with 80 mg/kg ketamine and 12 mg/kg xylazine. An indwelling silastic catheter (SAI Infusion Technologies, Libertyville, Il) was inserted into the jugular vein and sutured in place as previously described [34,35]. Following catheter insertion, rats were mounted in a stereotaxic apparatus (Kopf Instruments, CA) for implantation of electrodes or viral vector infusion with fiber optic implantation. Bipolar stainless steel electrodes (P1 Technologies, Roanoke, VA) were cut to 7.3 mm and implanted in the nucleus accumbens shell at 1.0 mm A/P and ±3.0 mm M/L on a 17° angle, relative to bregma (Paxinos and Watson, 1997). Viral infusions and implantation of fiber optic targeting the nucleus accumbens shell were performed using the coordinates +1.0 mm A/P, ±3.0 mm M/L, −7.3 mm D/V on a 17° angle, relative to bregma (Paxinos and Watson, 1997). Bilateral infusions (1μl/side) of a Cre-dependent adeno-associated viral vector (AAV) expressing eYFP (AAV5-EF1a-DIO-eYFP-WPRE-hGH; Addgene) or a Cre-dependent AAV expressing ChR2 and eYFP (AAV5-EF1a-DIO-hChR2 (H134R)-eYFP-WPRE-hGH; Addgene) were delivered via Hamilton syringes (Reno, NV) into the nucleus accumbens shell. Viral vector delivery was immediately followed by placement of 200 μm fiber optic (Thor Labs, Newton, NJ) attached to stainless steel ferrules (Fiber Instrument Sales, Oriskany, NY) and cut to length to terminate just above the nucleus accumbens shell. Electrodes or ferrules were cemented in place by affixing dental acrylic to three stainless steel screws fastened to the skull. Rats recovered for at least seven days; catheters were flushed daily (Timentin, 0.93 mg/ml, in heparinized saline) during recovery and after each daily behavioral session.

### 2.4. Ex vivo slice preparation

Following extinction of cocaine self-administration, rats expressing AAV5-EF1a-DIO-eYFP-WPRE-hGH or AAV5-EF1a-DIO-hChR2(H134R)-eYFP-WPRE-hGH) in the nucleus accumbens shell were collected for whole-cell patch-clamp recordings as in our previous research [30,36-38] using methods designed to improve neuronal health in adult rodents [39,40]. Rats were deeply anesthetized with isoflurane, and then briefly perfused with ice-cold cutting solution containing (in mM): 92 N-methyl-d-glucamine (NMDG), 2.5 KCl, 1.2 NaH_2_P0_4_, 30 NaHCO_3_, 20 HEPES, 25 glucose, 5 sodium ascorbate, 2 thiourea, 3 sodium pyruvate, 10 MgSO_4_, and 0.5 CaCl_2_, saturated with carbogen (95 % O2/5 % CO2), pH adjusted to 7.4 with HCl. Rats were then decapitated and brains removed. Acute coronal slices of the nucleus accumbens (250 μm thick) were obtained using a VT1000S vibratome (Leica, Weltzar, Germany) in 4 °C cutting solution, then placed in a holding chamber of the same cutting solution, and incubated at 37 °C for 10–12 min. Slices were then transferred to a beaker of room temperature holding ACSF containing (in mM): 86 NaCl, 2.5 KCl, 1.2 NaH_2_PO_4_, 35 NaHCO_3_, 20 HEPES, 25 glucose, 5 sodium ascorbate, 2 thiourea, 3 sodium pyruvate, 1 MgCl_2_, and 2 CaCl_2_, saturated with carbogen, pH 7.3–7.4, osmolarity 305–315 mOsm. Slices were allowed to recover for at least 45 min before performing recordings.

### 2.5. Ex vivo electrophysiology

Slices were placed on a Nikon Eclipse FN1 upright microscope equipped for Differential Interference Contrast (DIC) infrared optics. The recording chamber was continuously perfused with oxygenated recording ACSF containing (in mM): NaCl 119, KCl 2.5, NaHCO_3_ 26, NaH_2_PO_4_ 1.2, glucose 12.5, HEPES 5, MgSO_4_ 1, CaCl_2_ 2, pH 7.3–7.4, osmolarity 305–315 mOsm. Solution was heated to 32 ± 1 °C using an automatic temperature controller (Warner Instruments, Holliston MA). Target brain regions were identified using a 5X objective and individual neurons were magnified with a 40X water immersion lens. Medium spiny neurons (MSNs) in the nucleus accumbens shell were identified by their morphology and low resting membrane potential (−70 to −85 mV), and D1DR- or D2DR-containing neurons were further identified by fluorescent expression with a GFP filter (Nikon). Recording pipettes were pulled from borosilicate glass capillaries (World Precision Instruments, Sarasota, FL) to a resistance of 4.0–5.3 MΩ when filled with intracellular solution. The intracellular solution contained the following (in mM) potassium gluconate 145, KCl 2.5, NaCl 2.5, BAPTA 0.1, HEPES 10, l-glutathione 1.0, sodium phosphocreatine 7.5, Mg-ATP 2.0, and Tris-GTP 0.25, pH 7.2–7.3 with KOH, osmolarity 285–295 mOsm. All recordings were performed in whole-cell voltage-clamp or current-clamp mode using a MultiClamp 700B amplifier and Digidata 1440A Digitizer (Molecular Devices, San Jose, CA). A concentric bipolar stimulating electrode (FHC, Bowdoin, ME) was placed over axon fibers in close proximity to the recorded neuron. These projections were stimulated using 0.1 ms pulses (at 0.05 Hz) delivered from an isolated current stimulator (A-M instruments; Digitimer Ltd, Hertfordshire, England), and the evoked excitatory postsynaptic currents (EPSCs) were recorded. For paired pulse delivery, each pulse was separated by a 50 ms inter-pulse interval. For experiments involving optical stimulation, ChR2+ neurons were directly stimulated using blue light (473 nm) from either a DPSS laser (OptoEngine, Midvale, UT) or an external 4 channel LED driver (Thor Labs, Newton NJ). The LED was coupled to a fluorescent microscope port, allowing illumination of the slice through the objective, placed immediately above the cell. The laser/LED intensity was adjusted to the minimal amplitude (typically 1 mW or 10–50 mA) necessary to elicit action potential firing with a single 5 ms pulse. Following collection of baseline EPSCs (at least 10 min of stable responding), neurons received 10 min of 12 Hz stimulation (delivered either electrically or optically). A stimulation duration of 10 min corresponds with the highest period of responding in a cocaine-primed reinstatement session and limiting the duration of stimulation helped preserve the health of patched neurons. EPSCs were continuously recorded at 0.05 Hz for ≥ 40 min. The initial baseline amplitudes of EPSCs were compared to the EPSC amplitude following the DBS stimulation protocol (averaged over 10 mins pre-stim and last 10 min post-stim). All analyses of intracellular recordings were performed with Clampfit 10 (Molecular Devices). The experimenter was blind to treatment conditions during all electrophysiological recordings and analyses. For all experiments, series resistance was 10–25 MΩ, uncompensated, and monitored continuously during recording. Cells with a change in series resistance beyond 20 % were not accepted for data analysis. Synaptic currents were filtered at 3 kHz, amplified 5 times, and then digitized at 20 kHz. Picrotoxin (100 μM; dissolved in DMSO) was included in the recording solution to inhibit GABA_A_ receptor-mediated currents in all experiments.

### 2.6. Cocaine self-administration, extinction, and reinstatement

Rats were placed in operant conditioning chambers (Med-Associates, East Fairfield, VT) and allowed to press the active lever for intravenous cocaine infusions (0.25 mg of cocaine in 59 μL of saline, infused over 5 s, maximum of 30 infusions/session); inactive lever responses produced no scheduled consequences. Rats began training on a fixed ratio 1 (FR1) schedule of reinforcement. When stable responding was achieved on the FR1 schedule (less than 15 % variation over three consecutive days), rats were switched to an FR5 schedule. A 20 s timeout period during which active lever responses had no scheduled consequences followed each cocaine infusion. After 21 days of cocaine self-administration sessions, rats underwent an extinction phase during which cocaine was replaced with 0.9 % saline. Daily 2-hour extinction sessions were conducted until active lever responding was <20 % of the responses averaged over the last three days of cocaine self-administration. Rats designated for *ex vivo* slice electrophysiology were evaluated at this timepoint to recapitulate the neuronal status of rats immediately prior to a reinstatement session. Cocaine seeking was reinstated by non-contingent administration of cocaine (10 mg/kg, i.p.) immediately prior to the initiation of the reinstatement session, during which satisfaction of the response requirement is not reinforced. Each reinstatement test day was followed by extinction sessions until responding was again <20 % of the responses achieved during self-administration across two consecutive sessions.

### 2.7. Deep brain stimulation

Each rat underwent multiple cocaine-primed reinstatement sessions during which intra-accumbens shell DBS was delivered at 150 μA and 12 Hz, 60 Hz, 100 Hz, 130 Hz, or 0 μA (sham) in a within-subjects counterbalanced order to obviate any effect of test order. Alternating current with biphasic symmetrical pulses (60 μs pulse width) and a 150 μA stimulation intensity were used for all DBS frequencies. DBS was initiated concurrently with the onset of the reinstatement session and administered bilaterally and continuously throughout the 2-hour reinstatement session, as described previously [12-14,16].

### 2.8. Opto-DBS

Each rat underwent two reinstatement sessions, during which 473 nm light stimulation delivered at 12 Hz with a 5 ms pulse width or sham opto-DBS (patch cables attached but 0 mW delivered) was administered in a within-subjects counterbalanced fashion. Opto-DBS was administered continuously during the 1-hour reinstatement sessions, as described previously [30]. Light from a 473 nm laser (OptoEngine, Midvale, UT) was split by a rotary joint (Doric Lenses, Quebec, Canada) delivered bilaterally through 200 μm fiber optic patch cables (Thor Labs) connected to the implanted ferrules. Frequency was modulated by a Master 8 pulse generator (AMPI, Jerusalem, Israel) and laser output was calibrated to 1 mW of light in the accumbens to prevent any effect of heat.

### 2.9. Verification of AAV expression and fiber optic or electrode placement

After the completion of electrical DBS experiments, brains were fixed in 10 % formalin. Coronal sections (100 μm) were made on a vibratome (Technical Products International; St. Louis, MO) and electrode track marks were visualized under a light microscope. After the completion of opto-DBS experiments, rats were given an overdose of pentobarbital (100 mg/kg) and perfused intracardially with 0.9 % saline followed by 4 % paraformaldehyde. The brains were removed and coronal sections (40 μm) were collected for visualization on a confocal microscope (Leica Biosystems, Buffalo Grove, IL). Alternately, rats were decapitated and viral placement was visualized using NIGHTSEA^™^ DFP^™^ Dual Fluorescent Protein Excitation Flashlight with NIGHTSEA^™^ Barrier Filter Glasses (Electron Microscopy Services, Hatfield, PA). Rats lacking Cre-dependent eYFP fluorescence or with fluorescence and/or fiber optic or electrode placement outside of the nucleus accumbens shell were excluded from subsequent data analysis.

### 2.10. Statistical analysis

Statistical analysis was performed in Prism 9.0 with alpha set at *p*<0.05. Rats that maintained catheter patency throughout the duration of cocaine self-administration were included in behavioral analyses. Only rats with verified nucleus accumbens shell placement for AAV infusions alone or with fiber optic or electrodes were included in behavioral or electrophysiological experiments. The effect of DBS on the reinstatement of cocaine seeking was assessed by two-way repeated measures ANOVA with stimulation and lever as factors. Dunnett’s preplanned comparison was used to compare each stimulation frequency to sham (0 mW) for electrical DBS. Bonferroni post-hoc analysis was used for opto-DBS. Electrophysiology data were analyzed by two-tailed paired *t*-test (post-stim vs. pre-stim), except for optical stimulation experiments which included rats that expressed either eYFP or ChR2 and were analyzed by two-way mixed measures ANOVA with the between-subjects factor of vector (eYFP vs. ChR.2) and within-subjects factor of stimulation (pre-stim vs. post-stim) followed by Bonferroni post-hos analysis. Cocaine infusions earned by D1DR-Cre and D2DR-Cre rats for each experiment were analyzed by two-tailed unpaired *t*-test.

## 3. Results

### 3.1. Nucleus accumbens shell DBS attenuates cocaine seeking in male rats across a range of frequencies

**Fig. 1.**
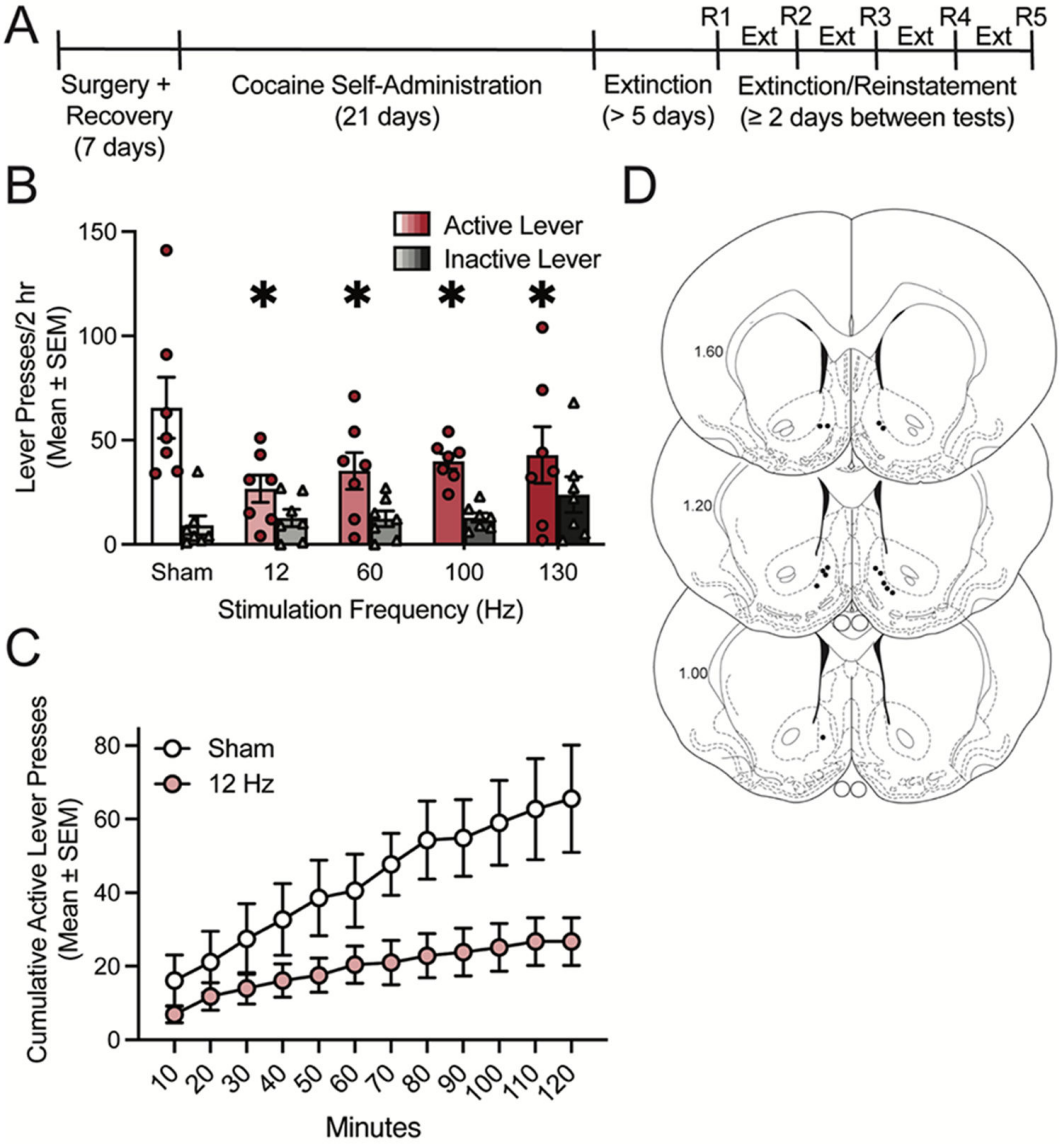
Deep brain stimulation of the nucleus accumbens shell attenuates cocaine seeking in male rats across a range of stimulation frequencies. A) Timeline of experiment. B) Bars show total responding (mean ± SEM) overlaid with active lever presses (circles) and inactive lever presses (triangles) for individual rats, with each rat receiving sham and each DBS frequency in a within-subjects design. Each DBS frequency significantly attenuated active lever responding in a cocaine-primed reinstatement test (*n* = 7). *p<0.05 vs. sham (0 μA). C) Time course of cumulative active lever presses throughout cocaine-primed reinstatement sessions where rats received sham stimulation or 12 Hz DBS. D) Electrode placements from the nucleus accumbens shell (dark circles). Values are in millimeters, relative to bregma.

Male Sprague Dawley rats were allowed to self-administer cocaine for 21 days on an FR1–5 schedule, as described previously [12,13,16,30] and as shown in the experimental timeline (Fig. 1A). Following cocaine self-administration training and extinction of lever responding, cocaine seeking was assessed in cocaine-primed reinstatement sessions during which rats received DBS at 12 Hz, 60 Hz, 100 Hz, and 130 Hz as well as sham stimulation (0 μA) in a within-subjects counterbalanced design. Repeated measures two-way ANOVA showed that there was no significant main effect of DBS (F_4,24_=1.66, *p* = 0.1917), a significant main effect of lever (F_1,6_ = 19.82, *p* = 0.0043), and a significant DBS x lever interaction (F_4,24_=4.12, *p* = 0.0111). Dunnett’s preplanned comparison revealed that there was a significant decrease in active lever responding, but not inactive lever responding, at each frequency of DBS compared to sham simulation (Fig. 1B). A time course of the cumulative active lever responding illustrates that the suppression of cocaine seeking by 12 Hz stimulation relative to sham stimulation is maintained throughout the 2-hour reinstatement sessions (Fig 1C). Electrode placements are shown in Fig. 1D. Taken together, these data indicate DBS attenuates cocaine-primed reinstatement of cocaine seeking in males similarly across a wide variety of stimulation frequencies. Because each stimulation frequency similarly attenuated cocaine seeking, we focused on the lowest frequency tested (12 Hz) for the subsequent studies.

### 3.2. Low frequency opto-DBS of accumbens shell D1DR- and D2DR-containing neurons attenuates cocaine seeking in male but not female rats

**Fig. 2.**
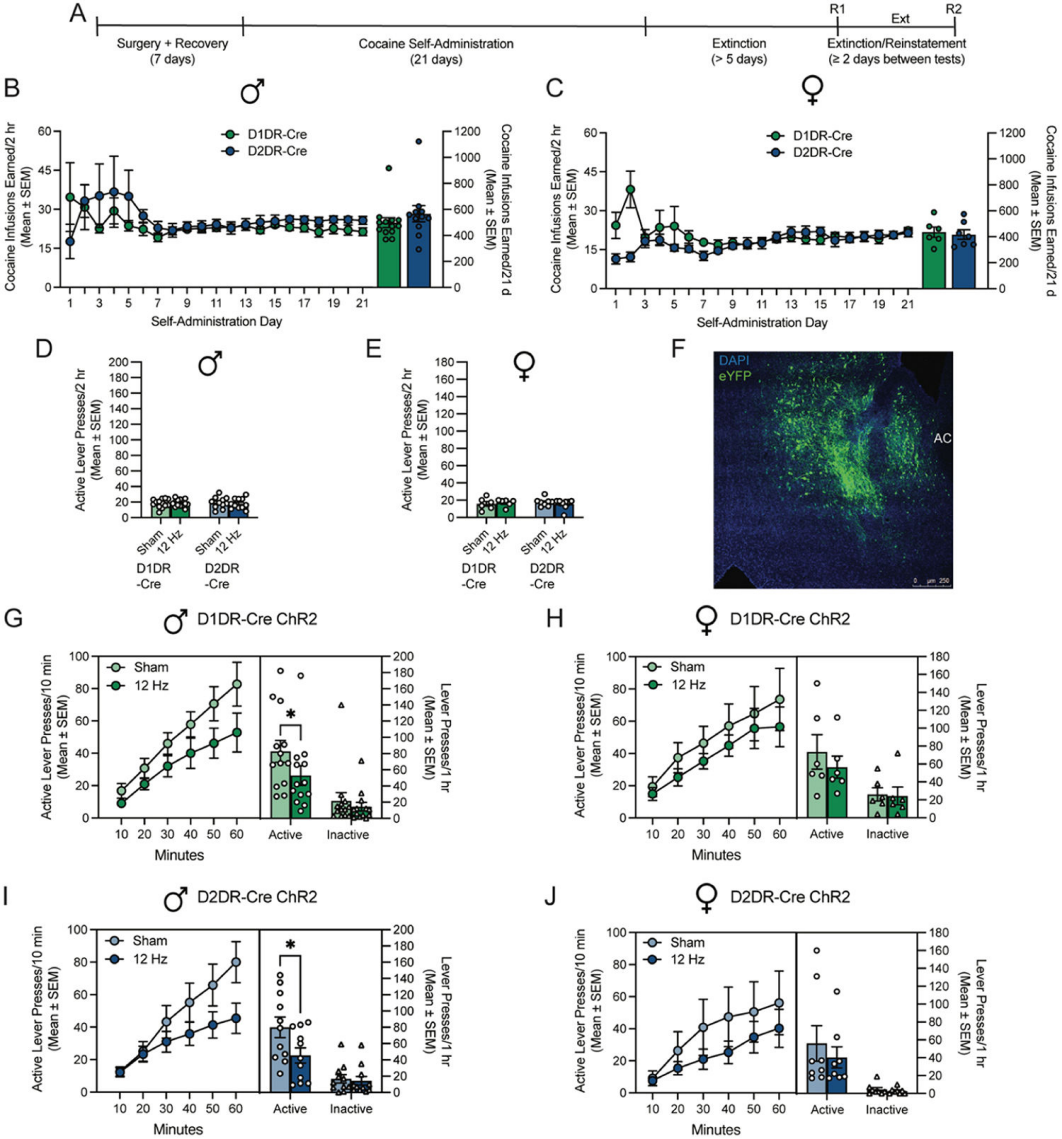
Low frequency opto-DBS in D1DR-containing and D2DR-containing neurons of the accumbens shell attenuates cocaine-primed reinstatement in male but not female rats. A) Experimental timeline. Mean (± SEM) cocaine infusions earned by B) male) and C) female D1DR-Cre and D2DR-Cre rats did not differ. Time courses show cumulative active lever presses during 1 hour reinstatement sessions. Bars show total responding overlaid with active lever presses (circles) and inactive lever presses (triangles) for individual rats, with each rat receiving sham and 12 Hz stimulation in a within-subjects design. C) Male and D) female rats extinguished active lever responding. There was no difference in the extinction baseline prior to reinstatement sessions with sham vs. 12 Hz optical stimulation. F) Representative image of eYFP expression in the nucleus accumbens shell. G) In male rats that expressed ChR2 in D1DR-containing neurons in the nucleus accumbens shell (*n* = 13), cocaine seeking was significantly attenuated by 12 Hz opto-DBS stimulation, relative to sham stimulation (*p<0.05). H) In female rats that expressed ChR2 in D1DR-containing neurons in the nucleus accumbens shell (*n* = 6), cocaine seeking did not differ when rats received sham stimulation or 12 Hz opto-DBS stimulation throughout the cocaine-primed reinstatement session. I) In male rats that expressed ChR2 in D2DR-containing neurons in the nucleus accumbens shell (*n* = 11), cocaine seeking was significantly attenuated by 12 Hz opto-DBS stimulation, relative to sham stimulation (*p<0.05). J) In female rats that expressed ChR2 in D2DR-containing neurons in the nucleus accumbens shell (*n* = 8), cocaine seeking did not differ when rats received sham stimulation or 12 Hz opto-DBS stimulation throughout the cocaine-primed reinstatement session.

The behavioral effect of low frequency opto-DBS in D1DR- and D2DR-expressing neurons to modulate cocaine seeking in male and female rats was explored. An experimental timeline is shown in Fig. 2A. Male (Fig. 2B) and female (Fig. 2C) rats expressing Cre-dependent ChR2 in DlDR-containing or D2DR-containing neurons self-administered cocaine for 21 days. There was no difference in total cocaine infusions earned between D1DR-Cre and D2DR-Cre males (t_1,22_=0.97; *p* = 0.3428; Fig. 2B) or females (t_1,11_=0.34; *p* = 0.7371; Fig. 2C). Lever responding was subsequently extinguished. There was no difference in extinction baseline, operationally defined as the average active lever response over the two sessions prior to each reinstatement test, between sham and 12 Hz optical stimulation tests in males (D1DR-Cre: t_1,12_=0.20, *p* = 0.8459; D2DR-Cre: t_1,10_=0.70, *p* = 0.4997; Fig. 2D) or females (D1DR-Cre: t_1,5_ = 0.33, *p* = 0.7554; D2DR-Cre: t_1,7_ = 0.76, *p* = 0.4696; Fig. 2E). Rats then received 12 Hz opto-DBS and sham stimulation throughout 1-hour cocaine-primed reinstatement tests in a within-subjects counterbalanced fashion. Only rats that reinstated above extinction baseline and expressed eYFP and ferrule placements in the accumbens shell were included in the analysis; a representative image showing eYFP expression in the nucleus accumbens shell is Fig. 2F. For all groups, active lever pressing during reinstatement sessions was significantly higher than responses during the immediately preceding extinction sessions. In male D1DR-Cre rats, two-way repeated measures ANOVA revealed a main effect of session (extinction vs. reinstatement) type (F_1,12_=25.65, *p* = 0.0003), a trend toward a main effect of stimulation (F_1,12_=4.12, *p* = 0.0650), and a trend toward a session type by stimulation interaction (F_1,12_=4.00, *p* = 0.0685). In male D2DR-Cre rats, two-way repeated measures ANOVA revealed a main effect of session type (F_1,12_=34.08, *p* = 0.0002), a main effect of stimulation (F_1,12_=5.98, *p* = 0.0345), and a trend toward a session type by stimulation interaction (F_1,12_=3.87, *p* = 0.0774). In female D1DR-Cre rats, two-way repeated measures ANOVA revealed a main effect of session type (F_1,5_ = 14.28, *p* = 0.0129), no main effect of stimulation (F_1,5_ = 0.69, *p* = 0.4454), and no session type by stimulation interaction (F_1,5_ = 1.03, *p* = 0.3579). In female D2DR-Cre rats, two-way repeated measures ANOVA revealed a main effect of session type (F_1,7_ = 5.69, *p* = 0.0485), no main effect of stimulation (F_1,7_ = 0.93, *p* = 0.3674), and no session type by stimulation interaction (F_1,7_ = 0.45, *p* = 0.5217). Thus, cocaine seeking was reinstated by a cocaine priming injection in all groups.

In rats that expressed ChR2 in nucleus accumbens shell D1DR-containing neurons, low frequency opto-DBS suppressed cocaine priming-induced reinstatement of drug seeking in male rats (Fig. 2G) but not female rats (Fig. 2H). In male D1DR-Cre rats, two-way repeated measures ANOVA revealed a trend toward a main effect of stimulation (F_1,12_=4.03, *p* = 0.0676), a main effect of lever (F_1,12_=23.95, *p* = 0.0004), and no lever by stimulation interaction (F_1,12_=3.12, *p* = 0.1029) on lever presses during the reinstatement sessions. Low frequency opto-DBS significantly attenuated responding on the active lever but not the inactive lever (Bonferroni post-hoc). In female D1DR-Cre rats, there was no main effect of stimulation (F_1,5_ = 0.75, *p* = 0.4263), a main effect of lever (F_1,5_ = 7.13, *p* = 0.0444), and no lever by stimulation interaction (F_1,5_ = 0.97, *p* = 0.3707) on lever presses during the reinstatement sessions.

Similarly, in rats that expressed ChR2 in nucleus accumbens shell D2DR-containing neurons, low frequency opto-DBS suppressed cocaine priming-induced reinstatement of drug seeking in male rats (Fig. 2I) but not female rats (Fig. 2J). In male D2DR-Cre rats, two-way repeated measures ANOVA revealed a trend toward a main effect of stimulation (F_1,10_=4.21, *p* = 0.0672), a main effect of lever (F_1,10_=61.20, *p*<0.0001), and a trend toward a lever by stimulation interaction (F_1,10_=4.89, *p* = 0.0515) on responses during the reinstatement sessions. Low frequency opto-DBS significantly attenuated responding on the active lever but not the inactive lever (Bonferroni post-hoc). In female D2DR-Cre rats, there was no main effect of stimulation (F_1,7_ = 0.75, *p* = 0.4150), a main effect of lever (F_1,7_ = 10.73, *p* = 0.0136), and no lever by stimulation interaction (F_1,7_ = 0.58, *p* = 0.4727) on lever presses during the reinstatement sessions. Collectively, these data indicate that low frequency optogenetic stimulation of D1DR- and D2DR-containing neurons attenuates cocaine priming-induced reinstatement in male rats, similar to that of electrical DBS stimulation (Fig. 1, [23]). However, low frequency optogenetic stimulation of D1DR-containing or D2DR-containing does not alter cocaine seeking in female rats, similar to prior work with high frequency optogenetic stimulation [30].

### 3.3. Low frequency electrical stimulation evokes long term potentiation in D1DR-MSNs and D2DR-MSNs from naive male and female rats

**Fig. 3.**
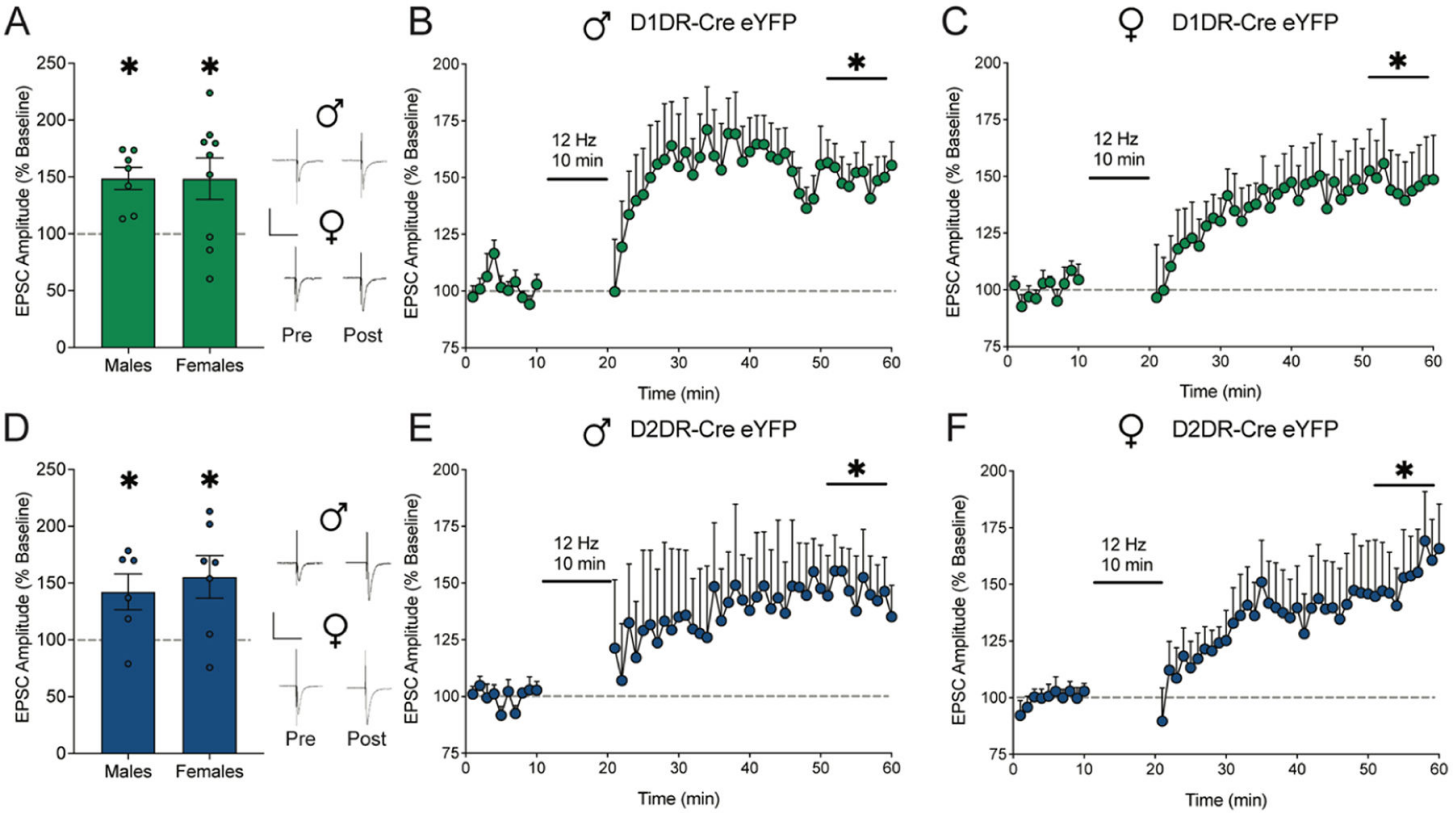
Low frequency electrical stimulation similarly elicits long term potentiation in D1DR-MSNs and D2DR-MSNs from cocaine naive male and female rats. A) EPSC amplitude averaged over the last 10 mins post-stimulation relative to 10 mins pre-stimulation baseline and full time course data for B) male (*n* = 7 cells/4 rats) and C) female (*n* = 9 cells/5 rats) rats expressing eYFP in D1DR-MSNs. D) EPSC amplitude averaged over the last 10 mins post-stimulation relative to 10 mins pre-stimulation baseline and full time course data for E) male (*n* = 6 cells/3 rats) and F) female (*n* = 7 cells/4 rats) rats expressing eYFP in D2DR-MSNs. Electrical stimulation at 12 Hz evoked LTP in D1DR-MSNs and D2DR-MSNs from cocaine naive male and female rats. *p<0.05 last 10 mins post-stimulation vs. 10 mins pre-stimulation baseline by two-tailed paired t-test. Insets show representative traces. Scale is 500 pA over 50 ms.

*Ex vivo* slice electrophysiology experiments were performed to evaluate the mechanistic effects of low frequency electrical stimulation on neuronal firing and synaptic plasticity. We first performed these experiments in naive rats to establish whether there were baseline differences by MSN subtype or sex. The effect of 12 Hz electrical stimulation on synaptic plasticity in D1DR-MSNs and D2DR-MSNs was assessed in nucleus accumbens shell Cre-dependent eYFP-positive MSNs from cocaine naive male and female D1DR-Cre and D2DR-Cre rats. The 12 Hz stimulation protocol evoked long term potentiation (LTP) in D1DR-MSNs (Fig. 3A) from male (t_1,6_ = 4.99, *p* = 0.0025; Fig. 3B) and female (t_1,8_ = 2.65, *p* = 0.0291; Fig. 3C) D1DR-Cre rats as well as in D2DR-MSNs (Fig. 3D) from male (t_1,6_ = 2.68, *p* = 0.044; Fig. 3E) and female (t_1,6_ = 2.95, *p* = 0.0255; Fig. 3F) D2DR-Cre rats. Thus, low frequency electrical stimulation similarly evoked LTP across sex and MSN subtype in naive rats.

### 3.4. Low frequency electrical stimulation selectively evokes long term potentiation in D2DR-MSNs from cocaine-experienced male rats

**Fig. 4.**
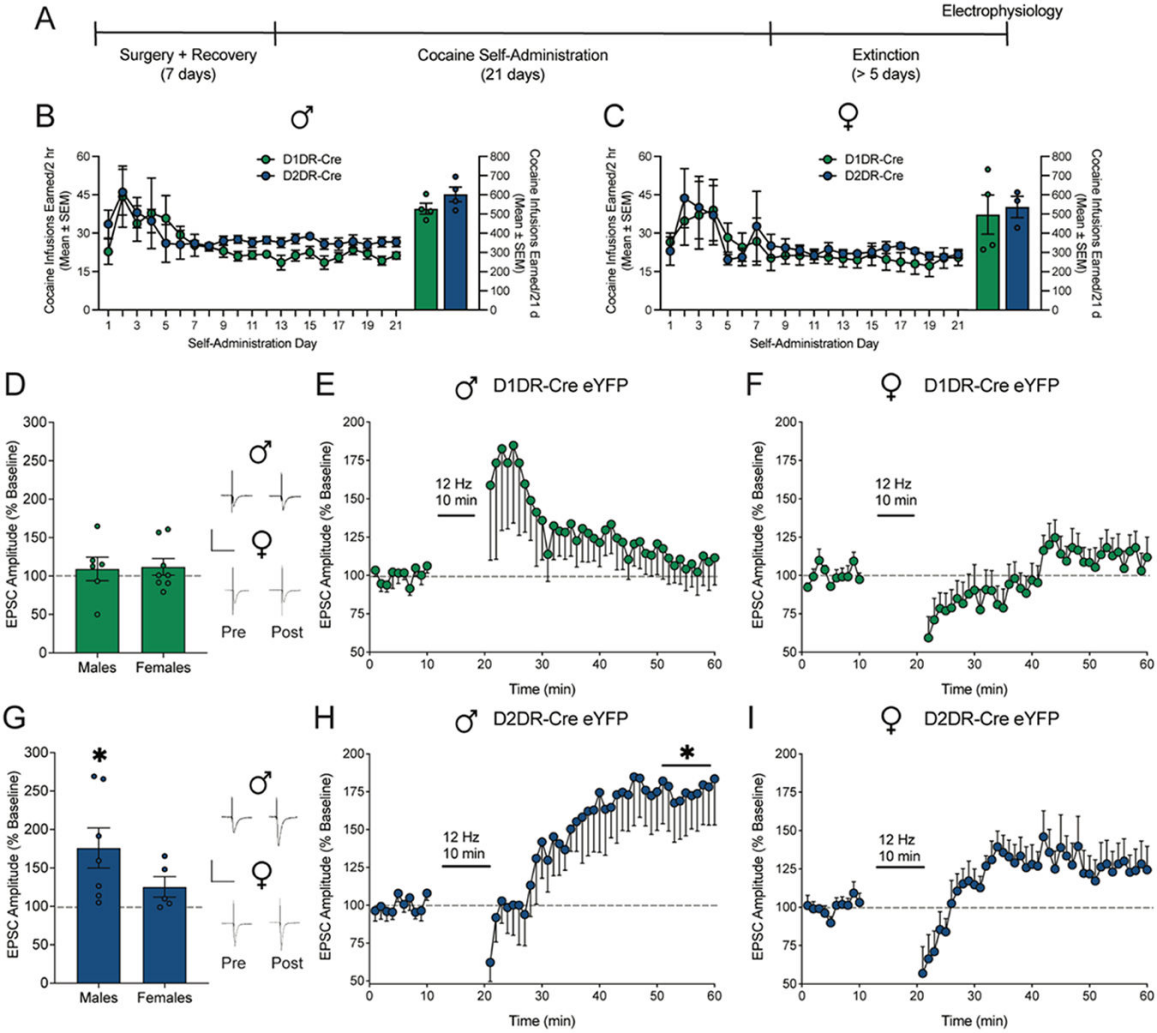
Low frequency electrical stimulation selectively elicits long term potentiation in D2DR-MSNs from cocaine-experienced male rats. A) Timeline of experiment. Mean (± SEM) cocaine infusions earned by B) male and C) female D1DR-Cre and D2DR-Cre rats. D) EPSC amplitude averaged over the last 10 mins post-stimulation relative to 10 mins pre-stimulation baseline and full time course data for E) male (*n* = 6 cells/4 rats) and F) female (*n* = 8 cells/4 rats) rats expressing eYFP in D1DR-MSNs. Electrical stimulation at 12 Hz did not evoke LTP in D1DR-MSNs from cocaine-experienced male and female rats. G) EPSC amplitude averaged over the last 10 mins post-stimulation relative to 10 mins pre-stimulation baseline and full time course data for H) male (*n* = 7 cells/4 rats) and I) female (*n* = 5 cells/3 rats) rats expressing eYFP in D2DR-MSNs. Electrical stimulation at 12 Hz selectively evoked LTP in D2DR-MSNs from cocaine experienced male, but not female, rats. *p<0.05 last 10 mins post-stimulation vs. 10 mins pre-stimulation baseline. Insets show representative traces by two-tailed paired t-test. Scale is 500 pA over 50 ms.

We next evaluated the effect of low frequency electrical stimulation on LTP in cocaine-experienced rats, as indicated in the experimental timeline (Fig. 4A). Male (Fig. 4B) and female (Fig. 4C) D1DR-Cre and D2DR-Cre rats were trained to self-administer cocaine for 21 days. There was no difference in cocaine infusions earned between D1DR-Cre and D2DR-Cre males (t_1,6_ = 1.574; *p* = 0.1666; Fig. 4B) or females (t_1,5_ = 0.31; *p* = 0.77; Fig. 4C). Lever pressing behavior was subsequently extinguished to criterion and to match the timepoint at which rats in behavioral experiments would undergo reinstatement testing. Slices from the nucleus accumbens shell were collected for electrophysiological recordings the next day.

The effect of 12 Hz electrical stimulation on long term potentiation was assessed in D1DR-MSNs and D2DR-MSNs labeled by Cre-dependent eYFP in D1DR-Cre and D2DR-Cre rats. Low frequency electrical stimulation failed to elicit LTP in D1DR-MSNs (Fig. 4D) from male (t_1,5_ = 0.61, *p* = 0.5705; Fig. 4E) or female (t_1,7_ = 1.09, *p* = 0.3107; Fig. 4F) cocaine-experienced rats. Failure to induce LTP may reflect cocaine-mediated potentiation in D1DR-MSNs [41], which could impede the ability of electrical stimulation to further augment plasticity. Alternately, cocaine has been shown to prevent LTP in D1DR-MSNs [41], which may not be reversed under these stimulation parameters. However, in D2DR-MSNs from cocaine-experienced rats (Fig. 4G), 12 Hz electrical stimulation significantly evoked LTP in males (t_1,6_ = 1.91, *p* = 0.027; Fig. 4H) but not females (t_1,4_ = 1.90, *p* = 0.1306; Fig. 4I). Cocaine has been shown to occlude plasticity within D2DR-MSNs [42,43], and these data suggest 12 Hz stimulation restores LTP within this subpopulation. These data indicate low frequency electrical stimulation selectively elicits LTP in D2DR-MSNs from male cocaine-experienced rats.

### 3.5. Low frequency optical stimulation selectively evokes long term potentiation in D2DR-MSNs from cocaine-experienced male rats

**Fig. 5.**
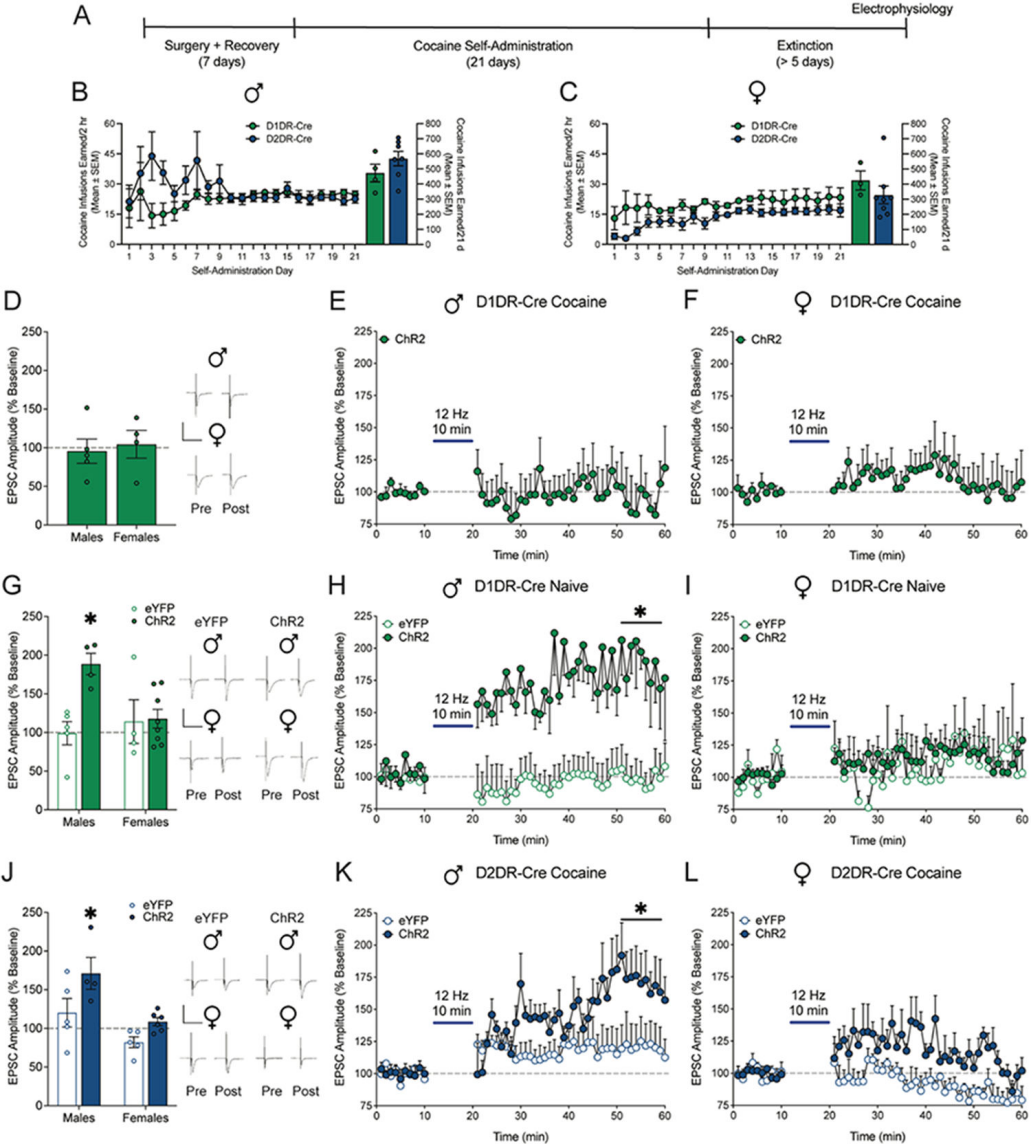
Low frequency optical stimulation selectively elicits long term potentiation in D2DR-MSNs from cocaine-experienced male rats. A) Timeline of experiment. Mean (± SEM) cocaine infusions earned by B) male and C) female D1DR-Cre and D2DR-Cre rats. D) EPSC amplitude averaged over the last 10 mins post-stimulation relative to 10 mins pre-stimulation baseline and full time course data for cocaine-experienced E) male (*n* = 5 cells/4 rats) and F) female (*n* = 4 cells/3 rats) rats expressing ChR2 in D1DR-MSNs. Optical stimulation at 12 Hz did not evoke LTP in D1DR-MSNs from cocaine-experienced male and female rats. G) EPSC amplitude averaged over the last 10 mins post-stimulation relative to 10 mins pre-stimulation baseline and full time course data for cocaine naive H) male (eYFP *n* = 5 cells/4 rats; ChR2 *n* = 4 cells/3 rats) and I) female (eYFP *n* = 4 cells/3 rats; ChR2 *n* = 8 cells/5 rats) rats expressing eYFP or ChR2 in D1DR-MSNs. Optical stimulation at 12 Hz selectively evoked LTP in D1DR-MSNs from cocaine experienced male, but not female, rats expressing ChR2. There was no change from baseline in male or female controls that expressed eYFP alone. J) EPSC amplitude averaged over the last 10 mins post-stimulation relative to 10 mins pre-stimulation baseline and full time course data for cocaine-experienced K) male (eYFP *n* = 5 cells/4 rats; ChR2 *n* = 4 cells/3 rats) and L) female (eYFP *n* = 5 cells/3 rats; ChR2 *n* = 6 cells/4 rats) rats expressing eYFP or ChR2 in D2DR-MSNs. Optical stimulation at 12 Hz selectively evoked LTP in D2DR-MSNs from cocaine experienced male, but not female, rats expressing ChR2. There was no change from baseline in male or female controls that expressed eYFP alone. *p<0.05 last 10 mins post-stimulation vs. 10 mins pre-stimulation baseline by Bonferroni post-hoc analysis. Insets show representative traces. Scale is 500 pA over 50 ms.

Electrical stimulation influences multiple cell types, perhaps differentially. To examine specific effects on accumbens output neurons, we used selective optogenetic low frequency stimulation of accumbens shell D1DR-MSNs and D2DR-MSNs. Fig. 5A shows an experimental timeline. Male (Fig. 5B) and female (Fig. 5C) D1DR-Cre and D2DR-Cre rats were trained to self-administer cocaine for 21 days. There was no difference in cocaine infusions earned between D1DR-Cre and D2DR-Cre males (t_1,9_ = 1.24; *p* = 0.2455; Fig. 5B) or females (t_1,9_ = 0.92; *p* = 0.3857; Fig. 5C).

The effect of 12 Hz optogenetic stimulation on long term potentiation was assessed in D1DR-MSNs or D2DR-MSNs from rats which were trained to self-administer cocaine and lever responding was subsequently extinguished. In D1DR-MSNs (Fig. 5D), 12 Hz optical stimulation did not evoke LTP in male (t_1,4_ = 0.26, *p* = 0.8090; Fig. 5E) or female (t_1,3_ = 0.24, *p* = 0.8241; Fig. 5F) cocaine-experienced D1DR-Cre rats which expressed Cre-dependent ChR2.

To verify that this failure of optical stimulation to elicit LTP is dependent upon cocaine experience rather than a differential effect of optical vs. electrical stimulation, the effect of 12 Hz optical stimulation was assessed in cocaine naive D1DR-Cre rats which expressed Cre-dependent eYFP or ChR2. Similar to low frequency electrical stimulation, 12 Hz optical stimulation evoked LTP in male (Fig. 5G, H) but not female D1DR-Cre rats expressing ChR2 (Fig. 5G, I). Mixed measures two-way ANOVA with stimulation (vs. baseline) and vector (eYFP vs. ChR2) as factors was used to assess the effect of 12 Hz optical stimulation on LTP. In male cocaine naive D1DR-Cre rats, there was a significant main effect of stimulation (F_1,7_ = 16.24, *p* = 0.0050), a main effect of vector (F_1,7_ = 19.52, *p* = 0.0031), and a significant stimulation x vector interaction (F_1,7_ = 17.02, *p* = 0.0044). Bonferroni post-hoc analysis showed that EPSC amplitude was significantly higher following 12 Hz optical stimulation in male D1DR-Cre rats that expressed ChR2. In female cocaine naive D1DR-Cre rats, there was no main effect of stimulation (F_1,10_=1.47, *p* = 0.2539), no main effect of vector (F_1,10_=0.028, *p* = 0.8705), and no stimulation x vector interaction (F_1,10_=0.014, *p* = 0.9081). There was no effect of optical stimulation in eYFP controls (Fig. 5G-I). Thus, the inability of 12 Hz optical stimulation to elicit LTP in D1DR-MSNs is driven by a history of cocaine self-administration and not the failure of optical stimulation to evoke LTP in this population of neurons.

In D2DR-MSNs (Fig. 5J), 12 Hz optical stimulation selectively evoked LTP in male (Fig. 5K) but not female (Fig. 5L) cocaine-experienced D2DR-Cre rats which expressed Cre-dependent ChR2, but not eYFP. In male cocaine-experience D2DR-Cre rats, there was a main effect of stimulation (F_1,7_ = 9.54, *p* = 0.0176), a main effect of vector (F_1,7_ = 9.52, *p* = 0.0177), and a significant stimulation x vector interaction (F_1,7_ = 9.80, *p* = 0.0209). Bonferroni post-hoc analysis showed that EPSC amplitude was significantly higher following 12 Hz optical stimulation in male D2DR-Cre rats that expressed ChR2 but not eYFP. In female cocaine-experience D2DR-Cre rats, there was no main effect of stimulation (F_1,9_ = 1.154, *p* = 0.3106), a main effect of vector (F_1,9_ = 9.26, *p* = 0.0140), and a stimulation x vector interaction (F_1,9_ = 10.15, *p* = 0.0111). There was no difference in EPSC amplitude following optical stimulation in female D2DR-Cre rats expressing ChR2 or eYFP. These results mirror the effects of electrical stimulation and suggest that low frequency optical stimulation selectively elicits LTP in D2DR-MSNs from male cocaine-trained rats.

## 4. Discussion

The present findings demonstrated that low frequency DBS of the nucleus accumbens shell attenuated cocaine seeking in male rats. Low frequency opto-DBS of D1DR- and D2DR-containing accumbens shell neurons similarly attenuated cocaine-primed reinstatement of cocaine seeking in male rats despite these subtype-specific differences in the induction of long-term synaptic plasticity. Low frequency opto-DBS of either subpopulation of neurons failed to influence cocaine seeking in female rats similar to our recent work using high frequency opto-DBS [30], which indicates that there may be important sex differences in the efficacy of accumbens shell DBS. While 12 Hz electrical stimulation elicited LTP in both D1DR-MSN and D2DR-MSN subtypes from naive male and female rats, a history of cocaine self-administration and extinction training occluded this plasticity such that low frequency stimulation only evoked LTP in D2DR-MSNs from male cocaine-experienced rats. Selective optical stimulation of a subpopulation of cells replicated this effect such that LTP was only produced in D2DR-MSNs from male cocaine-trained rats, although low frequency optical stimulation in D1DR-MSNs from cocaine naive male rats also was able to elicit LTP. The present results indicate that low frequency DBS may suppress cocaine seeking in males via actions on both D1DR-MSNs and D2DR-MSNs, although stimulation may exert differential effects on long-term synaptic plasticity in MSN subtypes.

We expected that selective 12 Hz stimulation of D2DR-containing neurons would attenuate cocaine seeking given that i) high frequency opto-DBS of D2DR-containing neurons but not D1DR-containing neurons suppressed cocaine reinstatement [30] and that ii) 12 Hz stimulation evoked LTP in D2DR-MSNs, but not cocaine-exposed D1DR-MSNs. Surprisingly, cocaine seeking was similarly attenuated by 12 Hz opto-DBS of both D1DR- and D2DR-containing neurons. Mimicking DBS with low frequency optogenetic stimulation of prefrontal cortex inputs onto accumbens D1DR-MSNs has previously been shown to block cocaine sensitization and produce long term depression (LTD) [42]. Optogenetic stimulation replicated the behavioral effects of high frequency DBS administered alone and co-administration of 12 Hz electrical DBS with a D1DR antagonist [29]. In contrast, cocaine occluded LTP evoked by high frequency stimulation in D1DR-MSNs that projected to the ventral pallidum [42]. A stimulation protocol that induced LTD in naive D1DR-MSNs rescued cocaine-occluded LTP in D1DR-MSN outputs and also blocked cocaine sensitization [42]. Although the protocol used in our prior study [30] exceeded the kinetic capacity of ChR2 and thus the effect of high frequency opto-DBS on plasticity within D1DR-MSNs is unclear, differences in D1DR-MSN synaptic plasticity evoked by high vs. low frequency optical and/or electrical stimulation could explain the discrepancy in the effect of these frequencies of stimulation on cocaine seeking. Future work might focus on varying stimulation parameters to reverse cocaine-mediated occlusion of LTP in D1DR-MSNs and exploring the effect of such DBS protocols on cocaine seeking. Together with the present results, these studies support the idea that activation of the D1DR-containing subpopulation of MSNs can attenuate cocaine-related behaviors under certain conditions, including specific stimulation parameters and within specific pathways [27,28]. It also is important to establish a causal link between DBS-mediated LTP rescue in D2DR-MSNs and attenuation of cocaine-seeking. These findings underscore the importance of understanding the subtype-specific effects of stimulation parameters to optimize the therapeutic effect of DBS for substance use disorders.

Electrical DBS delivered at high frequencies induces a wide range of neurophysiological changes, including activation [44,45], inhibition locally or via antidromic stimulation [46-49], and/or circuit-wide action on fibers of passage [49-53]. Mimicking DBS with optogenetics allows isolation of the effect of stimulation directly on nucleus accumbens shell MSNs and excludes the range of other effects of electrical DBS that are likely to impinge upon neuronal firing. Here we show that low frequency (12 Hz) electrical stimulation delivered directly to the nucleus accumbens shell under these parameters evoked LTP in both D1DR-MSNs and D2DR-MSNs in naive rats. In rats that had self-administered cocaine and subsequently undergone extinction of lever pressing to criterion, LTP was elicited by electrical and optical DBS only in D2DR-MSNs from male rats. It is notable that this experimental design, used to replicate the neuronal state of rats at the time of cocaine reinstatement sessions, differs from prior work using acute or repeated non-contingent cocaine delivery paradigms (e.g., [29,41,42]). A few studies have examined MSN subtype-specific aspects of accumbens synaptic plasticity after cocaine self-administration and withdrawal [54,55]; however, extinction learning following cocaine self-administration alters neuronal plasticity within accumbens MSNs [56-58] and may alter the effect of DBS on cocaine seeking. Caveats in drawing comparisons across our electrophysiology and behavior results include that rats evaluated for cocaine seeking during intra-accumbens shell DBS or opto-DBS underwent a longer stimulation period and also received an acute injection of cocaine to reinstate lever pressing. We have not yet established how acute re-exposure to cocaine might influence stimulation-evoked LTP.

The absence of an effect of low frequency opto-DBS on cocaine seeking in female rats replicates our prior work with high frequency opto-DBS [30]. It is important to note that the ability of electrical DBS to alter cocaine seeking in female rats, and whether the effect varies with different stimulation frequencies, has not yet been explored. In cocaine-experienced rats, 12 Hz optical stimulation was able to elicit LTP in D2DR-MSNs from cocaine-experienced male, but not female rats. The present study has not causally linked DBS-evoked LTP in MSN subtypes to cocaine seeking; however, sex differences in synaptic plasticity may underlie these behavioral differences. Optical stimulation did not evoke LTP in D1DR-MSNs from cocaine-experienced male or female rats, but only male rats showed attenuated cocaine seeking during opto-DBS of D1DR-MSNs. In naive rats, 12 Hz optical stimulation was only able to elicit LTP in D1DR-MSNs from male rats, despite the fact that LTP was observed in both sexes following electrical stimulation. This could point to a distinction between the ways in which electrical DBS, which includes indirect presynaptic effects not replicated by direct optical stimulation of accumbens shell MSNs, produces similar effects on LTP between males and females. Sex differences in excitability within the nucleus accumbens shell and key accumbens afferent (e.g., prefrontal cortex, ventral hippocampus) and/or efferent (e.g., ventral pallidum, ventral tegmental area) pathways may also govern the effect of accumbens shell stimulation. For example, testosterone regulates excitability in the ventral hippocampus to nucleus accumbens pathway via local androgen receptors, and female mice exhibit inherent increased excitability within this circuit [59]. Female rodents also exhibit inherent hyperexcitability relative to males in prefrontal cortex glutamate transmission [60] and estrous cycle-dependent differences in excitability in the nucleus accumbens core [61]. Inherent hyperexcitability in females may reduce the ability of DBS-like stimulation to evoke changes in plasticity and suppress cocaine seeking. Cocaine self-administration and extinction/reinstatement were performed identically across male and female rats in the present study; consideration of additional factors like gonadal hormones and estrous cycle or optimizing stimulation parameters (including frequency) may identify parameters in which DBS and opto-DBS effectively suppress cocaine seeking in females [62-64].

The present study investigated the effect of low frequency electrical DBS and opto-DBS on neuronal subpopulations within the shell subregion of the nucleus accumbens. High frequency electrical DBS attenuated cocaine-primed reinstatement of cocaine seeking in male rats when administered to the shell but not the core subregion of the nucleus accumbens [13]. However, it is possible that the regional specificity of DBS to suppress cocaine seeking could be frequency dependent, and future studies might assess whether low frequency stimulation of the accumbens core influences cocaine seeking behavior. Similarly, experiments to examine the effect of electrical DBS on cocaine seeking in female rats could include both shell and core subregions to determine if stimulation of these subregions differentially blunts cocaine seeking in a sex dependent manner. We also focused on cocaine-primed reinstatement given our previous findings that high frequency electrical DBS of the nucleus accumbens shell similarly attenuated cue-evoked reinstatement of seeking behavior for both cocaine and food [14]. Whether these behavioral effects similarly generalize to lower stimulation frequencies is an area for future study. It would be interesting to determine whether optical stimulation of D1DR-containing or D2DR-containing neuronal subpopulations would specifically suppress cue-evoked cocaine seeking vs. food seeking, which may be potentially useful for guiding treatment strategy.

## 5. Conclusions

Our work contributes to a growing literature which supports the use of DBS for substance use disorders [2-5]. The present study indicates that lower DBS frequencies are similarly efficacious in the suppression of craving, although the mechanisms by which high vs. low frequency stimulation alter behavior may be distinct. Refinement of DBS approaches by altering frequencies of stimulation or targeting distinct mechanisms (e.g., plasticity within neuronal subtypes) may improve treatment outcomes. Our study also highlights dramatic sex differences in the ability of accumbens shell DBS to attenuate drug craving, which is an important consideration when translating results such as these to the clinic.

## Data Availability

Data will be made available on request.
